# Alirocumab inhibits atherosclerosis, improves the plaque morphology, and enhances the effects of a statin[S]

**DOI:** 10.1194/jlr.M051326

**Published:** 2014-10

**Authors:** Susan Kühnast, José W. A. van der Hoorn, Elsbet J. Pieterman, Anita M. van den Hoek, William J. Sasiela, Viktoria Gusarova, Anusch Peyman, Hans-Ludwig Schäfer, Uwe Schwahn, J. Wouter Jukema, Hans M. G. Princen

**Affiliations:** * The Netherlands Organization of Applied Scientific Research (TNO) - Metabolic Health Research, Gaubius Laboratory, Leiden, The Netherlands; † Department of Cardiology, Leiden University Medical Center, Leiden, The Netherlands; § Einthoven Laboratory for Experimental Vascular Medicine, Leiden University Medical Center, Leiden, The Netherlands; * Regeneron Pharmaceuticals Inc., Tarrytown, NY; †† Sanofi-Aventis Deutschland GmbH, Frankfurt am Main, Germany

**Keywords:** APOE*3Leiden.CETP mice, proprotein convertase subtilisin/kexin type 9, atorvastatin

## Abstract

Proprotein convertase subtilisin/kexin type 9 (PCSK9) inhibition is a potential novel strategy for treatment of CVD. Alirocumab is a fully human PCSK9 monoclonal antibody in phase 3 clinical development. We evaluated the antiatherogenic potential of alirocumab in APOE*3Leiden.CETP mice. Mice received a Western-type diet and were treated with alirocumab (3 or 10 mg/kg, weekly subcutaneous dosing) alone and in combination with atorvastatin (3.6 mg/kg/d) for 18 weeks. Alirocumab alone dose-dependently decreased total cholesterol (−37%; −46%, *P* < 0.001) and TGs (−36%; −39%, *P* < 0.001) and further decreased cholesterol in combination with atorvastatin (−48%; −58%, *P* < 0.001). Alirocumab increased hepatic LDL receptor protein levels but did not affect hepatic cholesterol and TG content. Fecal output of bile acids and neutral sterols was not changed. Alirocumab dose-dependently decreased atherosclerotic lesion size (−71%; −88%, *P* < 0.001) and severity and enhanced these effects when added to atorvastatin (−89%; −98%, *P* < 0.001). Alirocumab reduced monocyte recruitment and improved the lesion composition by increasing the smooth muscle cell and collagen content and decreasing the macrophage and necrotic core content. Alirocumab dose-dependently decreases plasma lipids and, as a result, atherosclerosis development, and it enhances the beneficial effects of atorvastatin in APOE*3Leiden.CETP mice. In addition, alirocumab improves plaque morphology.

Proprotein convertase subtilisin/kexin type 9 (PCSK9) is a serine protease involved in LDL metabolism (1). PCSK9, previously known as neutral apoptosis regulated convertase, is mainly expressed in the liver, kidney, and intestines (2, 3).

Besides familial hypercholesterolemia (FH) caused by *ldlr* mutations and familial defective apoB100 caused by *apob* mutations (1), autosomal dominant hypercholesterolemia can be caused by gain-of-function mutations in the *pcsk9* gene, now commonly referred to as FH3 (3, 4). Conversely, loss-of-function mutations of the *pcsk9* gene were associated with a reduction in LDL cholesterol (LDL-C) and protection against coronary heart disease (5, 6). In addition, familial hypobetalipoproteinemia related to loss-of-function mutations of *pcsk9* resulted in very low plasma levels of LDL-C, attributed to an increased clearance rate of LDL (7).

Several studies have confirmed that PCSK9 is responsible for targeting the LDL receptor (LDLR) for lysosomal degradation in the liver by preventing recycling of the receptor to the cell membrane after internalization of the LDL-bound LDLR (4). PCSK9 interacts with the LDLR on the cell membrane, after which the LDLR-PCSK9 complex is internalized and travels through the endosome to the lysosome for degradation (8). In a study in mice, adenovirus-mediated expression of PCSK9 increased plasma LDL-C levels, which was associated with decreased hepatic LDLR protein, although LDLR mRNA levels were unaffected (9). On the contrary, mice lacking PCSK9 have decreased plasma LDL-C as a result of increased hepatic LDLR levels (10). A recent study in wild-type, APOE^−/−^, and LDLR^−/−^ mice with or without expression of PCSK9 revealed a direct relationship between PCSK9 and atherosclerosis development, mainly mediated via the LDLR, and suggests that PCSK9 inhibition will be beneficial in reducing atherosclerosis (11).

Although statins remain the most effective treatment option for CVD, there remains a substantial persistent cardiovascular risk, and, despite statin treatment, some patients cannot reach the recommended LDL-C target (12, 13). Recent outcome studies and post hoc analyses indicate that therapeutic regimens that further lower LDL-C lead to further reductions in cardiovascular events (14–16), and, consequently, cholesterol management guidelines have evolved to more rigorous goals (17–19). The upregulation of the LDLR after statin treatment is accompanied by an upregulation of PCSK9, which in turn promotes LDLR degradation (20–22). In humans, the ∼35% to 50% decrease in LDL-C after atorvastatin treatment (10 to 40 mg) was accompanied by a ∼7% to 35% increase in circulating PCSK9 levels (21, 22). Inhibition of PCSK9 is, therefore, a potential novel strategy for treatment of CVD, specifically in combination with statin treatment. Several approaches to inhibit PCSK9, including monoclonal antibodies, gene silencing, and mimetic peptides, are currently being investigated (4). The anti-PCSK9 monoclonal antibody alirocumab is a lead compound in this class and is currently being tested in phase 3 clinical trials.

Alirocumab, also known as SAR236553/REGN727, is a fully human, monoclonal antibody that lowers plasma LDL-C in normocholesterolemic volunteers (23) and hypercholesterolemic patients on stable statin dose (24, 25). In patients with hypercholesterolemia, alirocumab in combination with low- and high-dose atorvastatin decreased LDL-C to a greater extent than titration to high-dose atorvastatin, and considerably more patients who received the combination treatments reached LDL-C goals of <100 mg/dl or <70 mg/dl compared with patients who received atorvastatin treatment alone (26). Phase 2 trials demonstrated reductions in LDL-C of 40% to 72% across a dose range of 50 to 150 mg administered every 2 weeks, and of 32% to 48% with doses 200 to 300 mg administered every 4 weeks (23–25).

The aim of this study was to investigate the effects of two dosages of alirocumab alone and in combination with atorvastatin on plasma lipids, atherosclerosis development, and lesion composition in APOE*3Leiden.CETP mice (27). This is a well-established model for hyperlipidemia and atherosclerosis with all features of familial dysbetalipoproteinemia (FD) in humans, which is characterized by accumulation of remnant lipoproteins and an increased VLDL cholesterol to LDL-C ratio (28). APOE*3Leiden mice have an impaired clearance of (V)LDL and increased TG levels and are thereby mimicking the slow clearance observed in humans, in contrast to normal wild-type mice, which have a very rapid clearance of apoB-containing lipoproteins (29). The lipoprotein profile in APOE*3Leiden.CETP mice reflects that of FD patients with a similar response to lipid-modifying therapies (30), including statins (31), fibrates (32), niacin (33), and cholesteryl ester transfer protein inhibitors (34). This is illustrated by a comparable reduction in cholesterol in all apoB-containing lipoprotein subfractions with statin treatment (35). We hypothesized that alirocumab alone could reduce progression of atherosclerosis and add to the atheroprotective effects of atorvastatin. Inhibition of atherosclerosis by atorvastatin in APOE*3Leiden.CETP mice has been observed previously (34, 36).

## METHODS

### Animals and experimental design

Ninety female APOE*3Leiden.CETP transgenic mice on a C57/bl6 background (9 to 13 weeks of age) (27) received a semisynthetic cholesterol-rich diet for a run-in period of 3 weeks. After matching based on body weight, plasma total cholesterol (TC), TG, and age, mice (n = 15 per group) received a Western-type diet (WTD) alone or were treated with two dosages of alirocumab (3 or 10 mg/kg) alone or in combination with atorvastatin (3.6 mg/kg/d) for 18 weeks, and an arm with atorvastatin alone was added. Alirocumab (provided by Regeneron) was administered via weekly subcutaneous injections. All animals were euthanized by CO_2_ inhalation. Livers and hearts were isolated to assess hepatic LDLR protein levels, lipid content, atherosclerosis development, and plaque composition. Animal experiments were approved by the Institutional Animal Care and Use Committee of The Netherlands Organization for Applied Research.

### Plasma lipids, lipoprotein analysis, and measurement of alirocumab levels

Plasma TC and TG were determined every 2 to 4 weeks, and average plasma TC and TG were calculated by total exposure over number of weeks. Lipoprotein profiles for TC were measured after lipoprotein separation by fast protein LC (FPLC) (27). Alirocumab levels were measured by a human Fc enzyme-linked immunosorbent assay.

### Hepatic LDLR protein levels

LDLR protein level was evaluated by Western blotting using goat anti-mouse LDLR and rabbit anti-goat HRP, or mouse anti-α-tubulin and horse anti-mouse HRP; blots were developed with West Femto Super Signal ECL (Thermo Scientific) and subjected to the Chemi-Doc-it imaging system. Intensities of protein bands were quantified using Image J software.

### Histological assessment of atherosclerosis

Hearts were isolated, fixed in formalin, and embedded in paraffin. They were then sectioned perpendicular to the axis of the aorta, starting within the heart and working in the direction of the aortic arch. Once the aortic root was identified by the appearance of aortic valve leaflets and smooth muscle cells (SMCs) instead of collagen-rich tissue, serial cross-sections (5 µm thick with intervals of 50 µm) were taken and mounted on aminopropyl-triethoxy-silane (APES)-coated slides These sections were stained with hematoxylin-phloxine-saffron (HPS) for histological analysis. For each mouse, atherosclerosis was measured in four subsequent cross-sections. Each section consisted of three segments. The average total lesion area per cross-section was then calculated (36, 37). For determination of lesion severity, the lesions were classified into five categories according to the American Heart Association classification (38): *0*) no lesion, *I*) early fatty streak, *II*) regular fatty streak, *III*) mild plaque, *IV*) moderate plaque, and *V*) severe plaque. The percentage of each lesion type was calculated, where type I–III lesions were classified as mild lesions, and type IV–V lesions were classified as severe lesions (36, 37). Total plaque load in the thoracic aorta (from the aortic origin to the diaphragm) was determined after Oil Red O staining for lipids as described previously (39).

In the aortic root, lesion composition was determined for the severe lesions (type IV–V) as a percentage of lesion area after immunostaining with anti-human α-actin (1:800; Monosan, Uden, The Netherlands) for SMCs and anti-mouse Mac-3 (1:25; BD Pharmingen, The Netherlands) for macrophages followed by sirius red staining for collagen. Necrotic area was measured in macrophage/collagen staining. (36, 37, 40). In each segment used for lesion quantification, the number of monocytes adhering to the endothelium and the numbers of T cells in the total aortic root area were counted after immunostaining with AIA 31240 antibody (1:1000; Accurate Chemical and Scientific, New York, NY) and CD3 (1:500; AbD Serotec, Oxford, UK), respectively. Rat anti-mouse CD54 antibody, GTX76543 (GeneTex Inc., San Antonio, TX) was used for immunostaining of intercellular adhesion molecule 1 (ICAM-1) (41).

### Statistical analysis

Significance of differences between the groups was calculated nonparametrically using a Kruskal-Wallis test for independent samples, followed by a Mann-Whitney *U* test for independent samples. Linear regression analyses were used to assess correlations between variables.

IBM SPSS Statistics 20 for Windows (SPSS, Chicago, IL) was used for statistical analyses. All groups were compared with the control group and with the atorvastatin group, and 3 mg/kg alirocumab was compared with 10 mg/kg alirocumab either with or without atorvastatin. Values are presented as means ± SD. Bonferroni-Holm’s method was used to determine the level of significance in the case of multiple comparisons. *P* values <0.05 were considered statistically significant. In figures, significant effects after correction for multiple comparisons are indicated by * to compare with the control group, † to compare with the atorvastatin group, and ‡ to compare 3 mg/kg alirocumab with 10 mg/kg alirocumab.

For the full descriptions of the methods used, please see supplementary Methods.

## RESULTS

### Alirocumab and atorvastatin monotreatment and their combination decrease plasma TC and TG in APOE*3Leiden.CETP mice

**Fig. 1.**
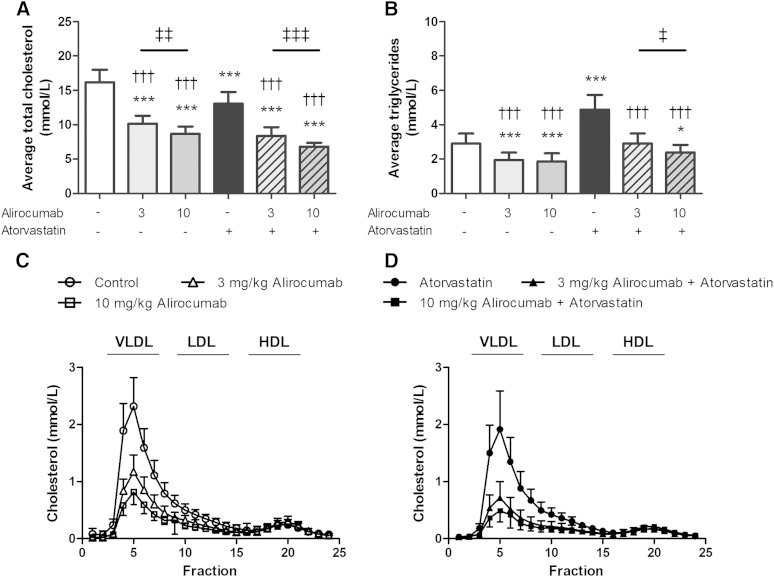
Effect of alirocumab, atorvastatin, and their combination on average plasma TC (A) and TG (B) levels as measured throughout the 18-week study. Lipoprotein profiles for cholesterol were assessed by FPLC lipoprotein separation to study effects of alirocumab alone (C) and in combination with atorvastatin (D). * *P* < 0.05, *** *P* < 0.001 as compared with control; ††† *P* < 0.001 as compared with atorvastatin; ‡ *P* < 0.05, ‡‡ *P* < 0.01, ‡‡‡ *P* < 0.001 for 3 mg/kg alirocumab compared with 10 mg/kg alirocumab (n = 15 per group).

Alirocumab binds both human and mouse PCSK9 with high affinity (*K_d_* = 0.58 nM and 2.6 nM, respectively, at pH 7.4 and 25°C) as determined by surface plasmon resonance (see supplementary data). Circulating alirocumab levels were detected in all groups administered alirocumab and ranged between 5 to 12 µg/ml (3 mg/kg dose) and 12 to 30 µg/ml (10 mg/kg dose) during the study. No immune response was observed as evidenced by stable efficacy throughout the study (see supplementary Fig. I). After 18 weeks of treatment, the APOE*3Leiden.CETP mice on a cholesterol-containing WTD (control group) reached average plasma TC and TG levels of 16.2 ± 1.8 mM and 2.9 ± 0.6 mM, respectively (**Fig. 1A, B**). Compared with the control, alirocumab decreased average plasma TC (−37%, *P* < 0.001; −46%, *P* < 0.001) and TG (−36%, *P* < 0.001; −39%, *P* < 0.001) and further decreased TC in combination with atorvastatin (−48%, *P* < 0.001; −58%, *P* < 0.001). Compared with atorvastatin, both combination treatments decreased TC (−36%, *P* < 0.001; −48%, *P* < 0.001) and TG (−39%, *P* < 0.001; −50%, *P* < 0.001) to a greater extent than atorvastatin alone. The (higher) reductions in TC (at the higher dose) after alirocumab alone (−14%, *P* < 0.01; 3 mg/kg alirocumab vs. 10 mg/kg alirocumab) and in combination with atorvastatin (−19%, *P* < 0.001; 3 mg/kg alirocumab + atorvastatin vs. 10 mg/kg alirocumab + atorvastatin) were dose-dependent and sustained during the study. TC reductions after alirocumab (Fig. 1C), atorvastatin, and their combination (Fig. 1D) were confined to non-HDLs.

### Alirocumab without and with atorvastatin decreases plasma lipids by reducing LDLR degradation

**Fig. 2.**
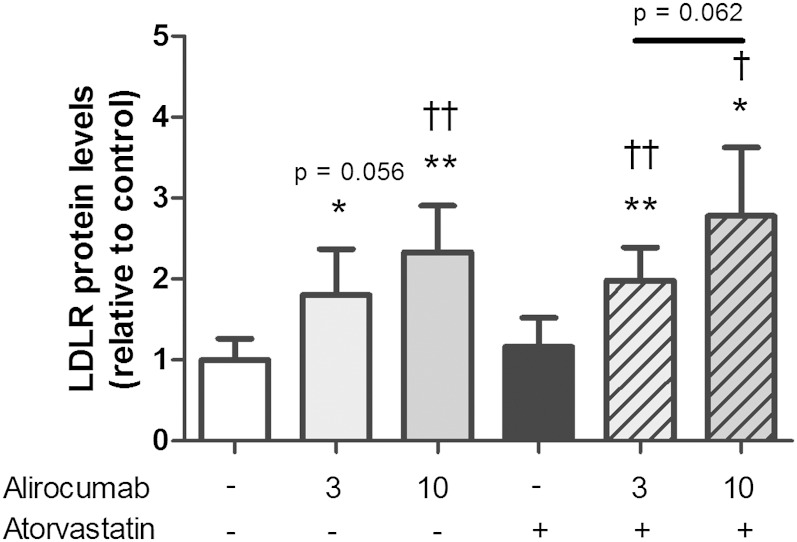
Effect of alirocumab, atorvastatin, and their combination on hepatic LDLR protein levels. * *P* < 0.05, ** *P* < 0.01 as compared with control; † *P* < 0.05, †† *P* < 0.01 as compared with atorvastatin (n = 8 per group).

Hepatic LDLR protein levels were measured to verify whether PCSK9 inhibition by alirocumab decreases plasma lipids by rescuing LDLR degradation (**Fig. 2**). Hepatic LDLR protein levels were increased after alirocumab treatment alone (+80%, *P* < 0.05; +133%, *P* < 0.01) and together with atorvastatin (+98%, *P* < 0.01; +178%, *P* < 0.05). Compared with atorvastatin alone, both the combination treatments increased LDLR protein levels to a greater extent (+71%, *P* < 0.01; +140%, *P* < 0.05). An inverse correlation between LDLR protein levels and plasma TC confirms the involvement of the LDLR in lowering of TC by alirocumab (*R*^2^ = 0.50, *P* < 0.001).

### Alirocumab dose-dependently reduces atherosclerosis development and enhances the atheroprotective effects of atorvastatin

**Fig. 3.**
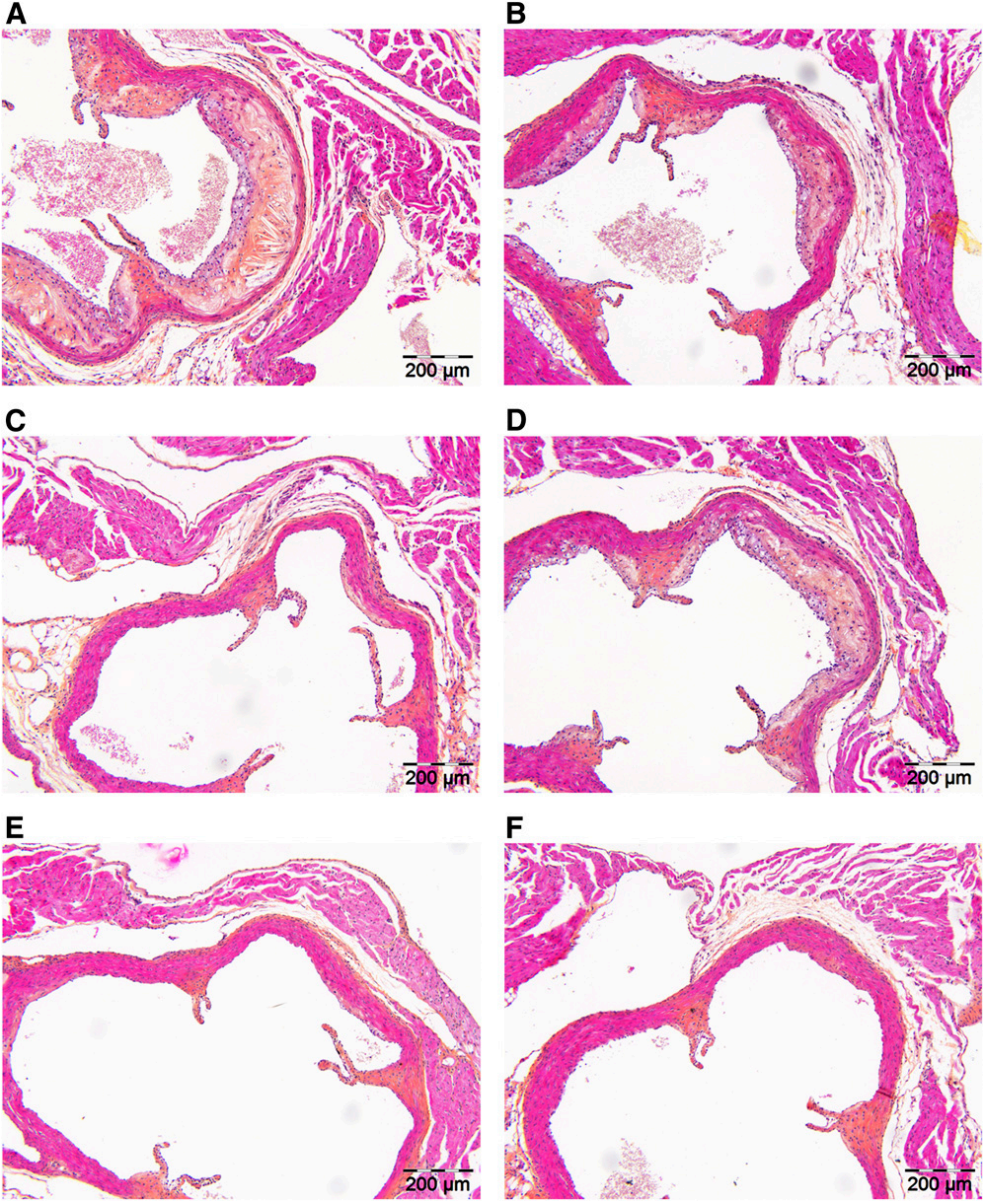
Effect of alirocumab, atorvastatin, and their combination on plaque morphology. Representative images of HPS-stained atherosclerotic lesions in a cross-section of the aortic root area for the control (A), 3 mg/kg alirocumab (B), 10 mg/kg alirocumab (C), atorvastatin (D), 3 mg/kg alirocumab + atorvastatin (E), and 10 mg/kg alirocumab + atorvastatin (F) groups, respectively, after 18 weeks of treatment.

**Fig. 4.**
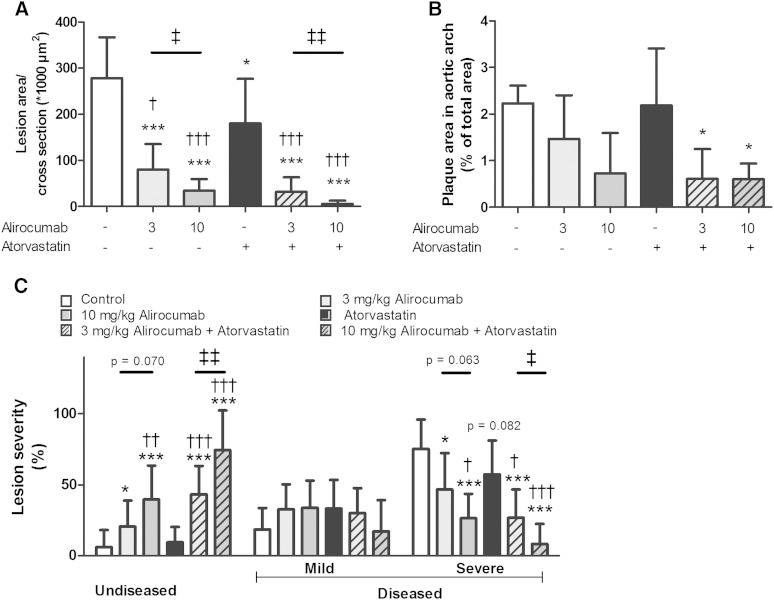
Effect of alirocumab, atorvastatin, and their combination on atherosclerosis development in aortic root and arch. After 18 weeks of treatment, the total lesion area per cross-section was assessed (A). The total plaque load in the aortic arch was analyzed after Oil Red O staining (B). Lesion severity was assessed and categorized as no lesions, mild lesions (type I–III), and severe (type IV–V) lesions (C). Data are expressed as percentage of the stained area. * *P* < 0.05, *** *P* < 0.001 as compared with control; † *P* < 0.05, †† *P* < 0.01, ††† *P* < 0.001 as compared with atorvastatin; ‡ *P* < 0.05, ‡‡ *P* < 0.01 for 3 mg/kg alirocumab compared with 10 mg/kg alirocumab (n = 15 per group in the root area and n = 6–7 in the arch).

Effects of alirocumab on atherosclerosis development in the absence and presence of atorvastatin were assessed in the aortic root and arch after 18 weeks of treatment. Representative images of atherosclerotic lesions as illustrated in **Fig. 3** show that alirocumab, atorvastatin, and their combination reduced lesion progression. To confirm a reduction in atherosclerosis development, we determined lesion area per cross-section (**Fig. 4A**) and calculated lesion severity (Fig. 4C). For the control group, total lesion area was 278 ± 89 × 10^3^ µm^2^ per cross-section. Alirocumab dose-dependently decreased atherosclerotic lesion size (−71%, *P* < 0.001; −88%, *P* < 0.001) and dose-dependently enhanced the effects of atorvastatin (−89%, *P* < 0.001; −98%, *P* < 0.001) as compared with the control. Mice treated with alirocumab alone and in combination with atorvastatin had more lesion-free sections and fewer severe (type IV–V) lesions compared with the control. Atorvastatin alone decreased lesion size (−35%, *P* < 0.05) and reduced severity to a lesser extent with no effect on the amount of undiseased segments, as counted by the number of undiseased segments per mouse and calculated as the percentage of undiseased segments per mouse. When compared with atorvastatin monotreatment, the combinations further decreased lesion size (−82%, *P* < 0.001; −97%, *P* < 0.001) and increased the amount of undiseased segments.

To evaluate the effect of alirocumab treatment on lesion development at another spot along the aorta prone to development of atherosclerosis, plaque surface in the aortic arch was measured (Fig. 4B). At this site, lesion development is delayed as compared with the aortic root (39). In line with the effects on atherogenesis in the aortic origin, both doses of alirocumab together with atorvastatin (−73%, *P* < 0.05; −73%, *P* < 0.05) reduced the total plaque area.

**Fig. 5.**
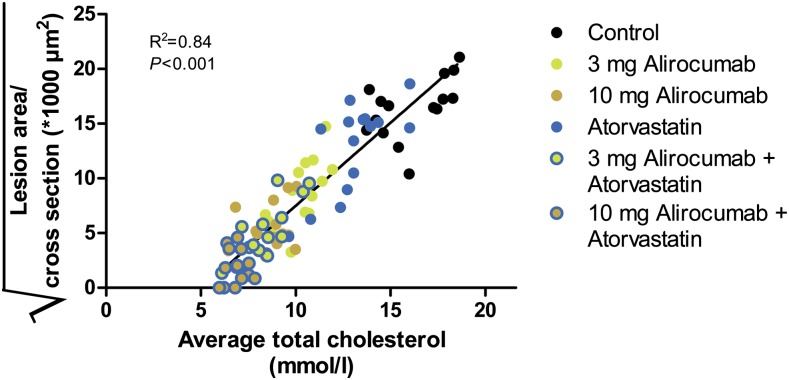
Correlation between average plasma TC and atherosclerotic lesion area. The square root of the lesion area was plotted against average TC. Linear regression analysis was performed.

We evaluated whether the antiatherogenic effect of alirocumab and atorvastatin could be explained by the reduction in plasma TC (**Fig. 5**). A strong correlation between plasma TC levels and atherosclerotic lesion area in the aortic root was observed (*R*^2^ = 0.84, *P* < 0.001; Fig. 5), indicating an important role of cholesterol in the development of atherosclerosis.

### Alirocumab improves plaque morphology

**Fig. 6.**
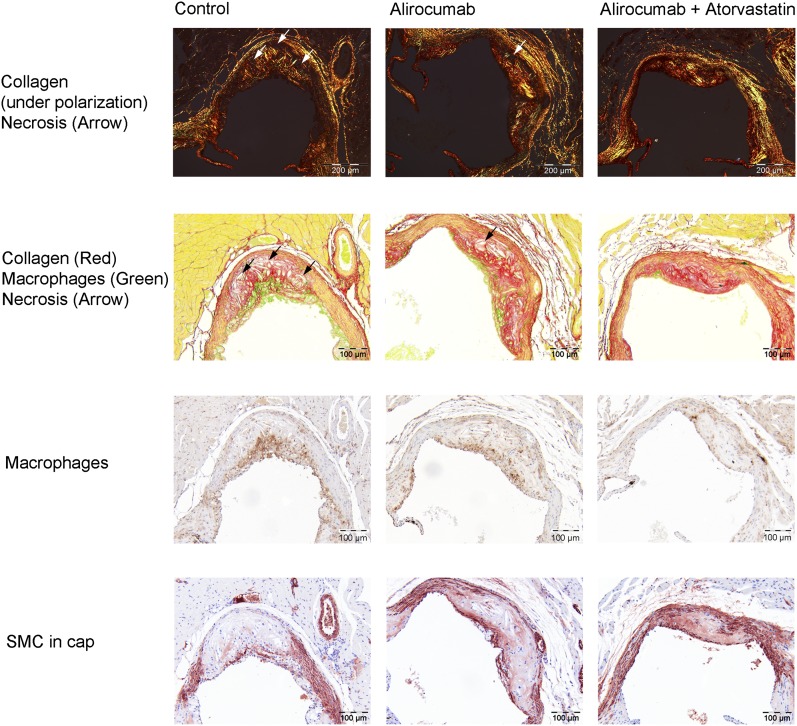
Effect of alirocumab, atorvastatin, and their combination on lesion composition. Representative images of immunostaining with Mac-3 for macrophages followed by sirius red staining for collagen and α-actin for SMCs for the control and after 18 weeks of treatment with alirocumab alone and in combination with atorvastatin.

**Fig. 7.**
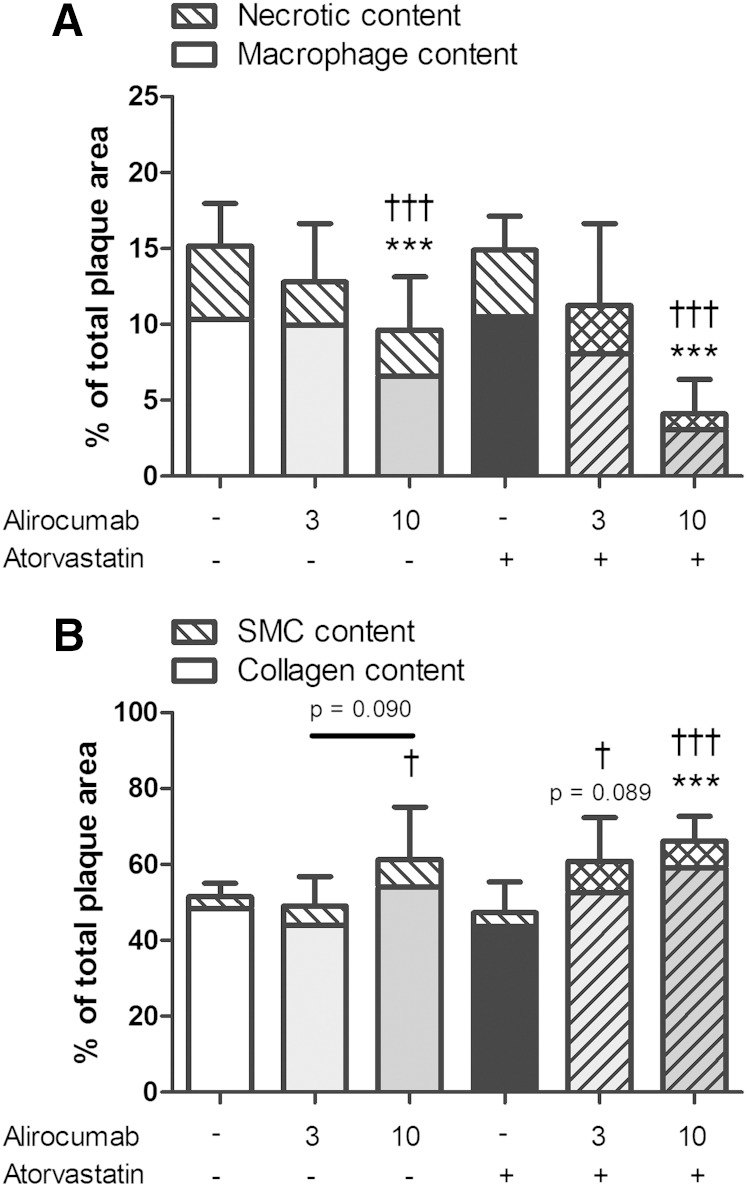
Effect of alirocumab, atorvastatin, and their combination on lesion composition. Macrophage and necrotic content, including cholesterol clefts, as proinflammatory factors (A), and SMCs and collagen content as fortifying factors (B), were determined in the severe (type IV–V) lesions after correcting for lesion size. ** *P* < 0.01, *** *P* < 0.001 as compared with control; † *P* < 0.05, †† *P* < 0.01, ††† *P* < 0.001 as compared with atorvastatin (n = 15 per group).

After investigating lesion morphology, we analyzed treatment effects on plaque composition in the severe lesions (type IV–V), as shown by representative images in **Fig. 6**. To illustrate that a proinflammatory plaque phenotype is not always dependent on the size of the lesions, we included representative images of similar size lesions for the control group and the alirocumab group. Lesion macrophage area and lesion necrotic core area (including cholesterol clefts) were quantified as proinflammatory factors (**Fig. 7A**), whereas SMCs in the fibrotic cap and collagen area were quantified as fortifying factors (Fig. 7B). All were expressed as a percentage of total lesion area. Lesions in the control group consisted of 10.3% macrophages, 4.8% necrotic core and cholesterol clefts, 3.1% SMCs in the cap, and 48.4% collagen. Alirocumab (10 mg/kg) alone and in combination with atorvastatin reduced the proinflammatory factors as compared with control (−37%, *P* < 0.001; −73%, *P* < 0.001) and with atorvastatin treatment (−35%, *P* < 0.001; −72%, *P* < 0.001). Fortifying factors were increased by 10 mg/kg alirocumab + atorvastatin as compared with control (+29%, *P* < 0.001) and dose dependently as compared with atorvastatin treatment (+29%, *P* < 0.05; +40%, *P* < 0.001).

### Alirocumab reduces monocyte and T-cell recruitment

**Fig. 8.**
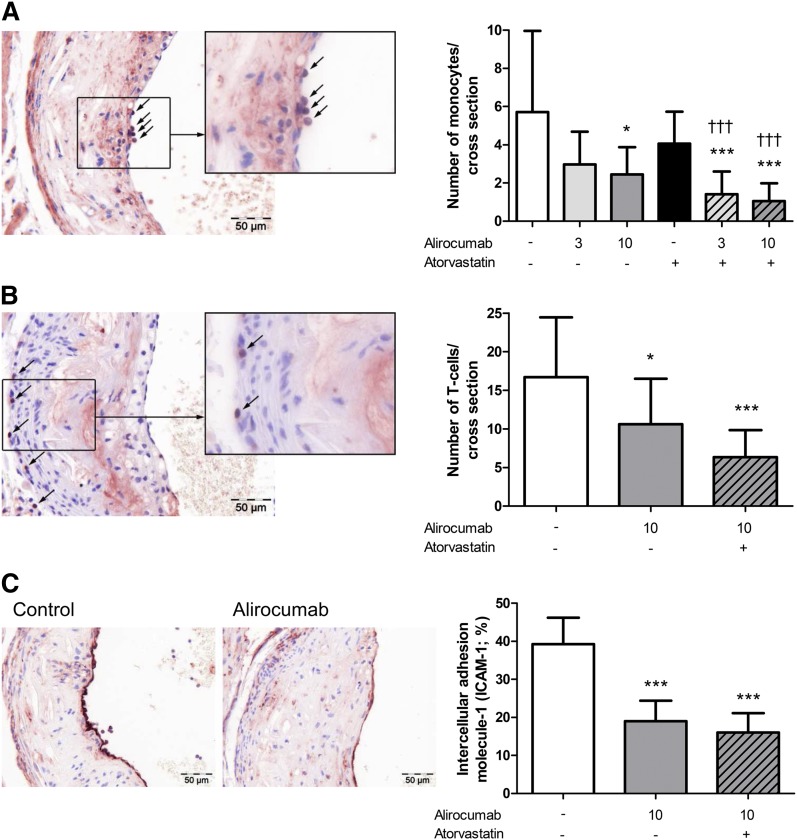
Effect of alirocumab, atorvastatin, and their combination on markers of vascular inflammation. The number of monocytes adhering to the endothelium (A) and the number of T cells in the aortic root area (B) were determined per cross-section. In addition, ICAM-1 was determined as percentage of the stained area (C). Representative images are included. * *P* < 0.05, *** *P* < 0.001 as compared with control; ††† *P* < 0.001 as compared with atorvastatin (n = 15 per group).

As a functional marker of vessel wall inflammation, the number of monocytes adhering to the activated endothelium (**Fig. 8A**) and the number of T cells in the aortic root area (Fig. 8B) were counted and calculated per cross-section (Fig. 7A). In the control group, 5.7 ± 4.2 adhering monocytes and 16.7 ± 7.7 T cells were present. When administered alone and together with atorvastatin, the higher dose of alirocumab (10 mg/kg) decreased the adhering monocytes (−57%, *P* < 0.05; −82%, *P* < 0.001) and the abundance of T cells (−37%, *P* < 0.05; −62%, *P* < 0.001). To further underline the mechanism by which alirocumab reduced monocyte adherence, we assessed endothelial ICAM-1 expression by immunohistochemistry (Fig. 8C). For the control, 39% of the endothelium was positive for ICAM-1 compared with 19% (*P* < 0.001) after 10 mg/kg alirocumab monotreatment and 16% (*P* < 0.001) when given in combination with atorvastatin. The reduction in monocyte adherence was, therefore, corroborated by a reduction in adhesion molecule expression in endothelial cells after alirocumab treatment alone and in combination with atorvastatin.

### Safety aspects of alirocumab

No effects on body weight (gain) and food intake were noted in any treatment group as compared with control (data not shown). The 10 mg/kg dose of alirocumab on top of atorvastatin treatment led to a reduction in liver weight as compared with the control group after 18 weeks of treatment (−20%, *P* < 0.05, respectively), whereas monotreatment did not have an effect on liver weight. Plasma aspartate transaminase and alanine transaminase were measured in all animals after 16 weeks of treatment; the data are summarized in supplementary Table I.

## DISCUSSION

The PCSK9 monoclonal antibody alirocumab has been shown to strongly lower LDL-C and non-HDL cholesterol (HDL-C) alone and on top of statin treatment (23–26) and is currently in phase 3 clinical development, which includes a large CVD outcome trial in hypercholesterolemic patients with relatively recent acute coronary syndrome treated with high-dose statins (42). It should be realized that the effectiveness of alirocumab on the cardiovascular end point will only be assessed in patients who also receive statins. Therefore, the present study was designed to investigate the effects of alirocumab per se on atherosclerosis development and in combination with atorvastatin. Taken together, we have shown that alirocumab dose-dependently decreases plasma cholesterol and reduces progression of atherosclerosis. Moreover, alirocumab improves lesion morphology and composition and enhances the beneficial effects of atorvastatin in APOE*3Leiden.CETP mice. This is the first study to show that a monoclonal antibody to PCSK9 reduces atherosclerosis development.

Rescue of LDLR from intracellular degradation was verified by an increase in hepatic LDLR protein levels after alirocumab treatment. Consequently, alirocumab decreased TC (−37% to −46%) and TG (−36% to −39%) by a reduction in non-HDLs. The dose-dependent reduction in TC after alirocumab treatment was enhanced in combination with atorvastatin (−48% to −58%). These results support an improvement in cholesterol management by adding alirocumab to statin treatment. The dose-dependent cholesterol-lowering effects in our study are in accordance with results from phase 1 and phase 2 clinical trials (23–26). In phase 1 trials, alirocumab administered as a single ascending dose (50 to 250 mg) in healthy subjects, and as multiple doses (50 to 150 mg) in statin-treated FH patients, decreased LDL-C by 33% to 46% and by 39% to 61%, respectively (23). Results from the latter study indicate an additive effect of alirocumab on statin treatment because similar reductions were observed with alirocumab monotherapy and in statin-treated patients.

In a phase 2 trial in patients with hypercholesterolemia, addition of 50 to 150 mg alirocumab every 2 weeks to 10 to 40 mg/d atorvastatin decreased LDL-C by 40% to 72% and TC by 23% to 45% (25). In a phase 2 trial in patients with FH, addition of 150 mg of alirocumab every 2 weeks to a stable dose of statin, with or without ezetimibe, decreased LDL-C by 68% and TC by 44% (24). A multicenter phase 2 trial confirmed these findings with a 66% to 73% reduction in LDL-C and a 41% to 47% reduction in TC when adding alirocumab to either 10 or 80 mg/d atorvastatin in hypercholesterolemic patients (26). In the present study, a similar additive cholesterol-lowering effect on top of atorvastatin treatment (−36% to −48% reductions in TC as compared with atorvastatin alone) was found. The clinical trials also provide evidence for additional modest reductions in TGs and modest increases in HDL-C. However, baseline TG levels were low in the latter studies, which may explain the larger effect on TG found in our study.

A higher (V)LDL clearance increases liver cholesterol exposure and may result in changes in hepatic cholesterol content and/or fecal excretion of cholesterol or, after its conversion, bile acids. However, alirocumab did not lead to hepatic lipid accumulation, whereas atorvastatin and the combination treatments significantly reduced cholesteryl ester content without changes in hepatic TG. Intriguingly, fecal output of bile acids and neutral sterols remained unchanged by the treatments (see supplementary data). In line with our data, full absence of PCSK9 was recently reported not to be associated with hepatic lipid accumulation or fecal excretion of cholesterol (43). However, contrasting data in PCSK9 ^−/−^ mice demonstrated increased LDL-C excretion via the transintestinal cholesterol excretion pathway and subsequently mildly increased fecal neutral sterol loss, with unfortunately no data on fecal bile acid loss (44). As opposed to PCSK9 inhibition by alirocumab in the present study, lack of PCSK9 was reported to increase fecal bile acid output (43). These data indicate that, despite the greater influx of cholesterol from the plasma compartment into the liver, hepatic cholesterol homeostasis is maintained, although the precise mechanism remains to be established.

The lipid-modifying effects of PCSK9 inhibition provide indications for an atheroprotective effect. This notion is supported by data from a study where mice expressing high levels of PCSK9 had significantly more aortic cholesterol ester accumulation and developed severe aortic lesions, compared with wild-type and PCSK9 knockout mice when fed an atherogenic diet (11). In the same study, no differences were found in LDLR-deficient mice expressing no, normal, or high PCSK9 levels, suggesting that PCSK9 modulates atherosclerosis mainly via the LDLR. However, to date, the atheroprotective effect of pharmacological PCSK9 inhibition has not been investigated. Our study demonstrates for the first time that inhibition of serum PCSK9 with the monoclonal antibody alirocumab decreases plasma lipid levels and as a result reduces atherosclerosis development, as evidenced by a reduction in atherosclerotic lesion size and severity in the aortic root area and arch. This dose-dependent inhibitory effect of alirocumab on lesion size was strongly enhanced in combination with atorvastatin, where a considerable number of animals did not develop any severe lesions. Although pleiotropic effects of statin treatment may contribute to the reduction in CVD risk (45), results from our study emphasize the importance of cholesterol lowering per se in treatment of CVD. In our study, the effects of alirocumab alone and in combination with atorvastatin on lesion area were mainly predicted by the reduction in plasma TC levels as illustrated by the strong association (*R*^2^ = 0.84) between TC levels and the lesion area.

In the present study, alirocumab, atorvastatin, and their combination reduced the circulating granulocytes/neutrophils and monocytes, in particular proinflammatory Ly6C^hi^ monocytes (see supplementary data). Ly6C^hi^ monocytes are proposed to be precursors of proinflammatory M1 macrophages (46), and studies in mice have shown that hypercholesterolemia induces Ly6C^hi^ monocytosis (47). In addition, alirocumab decreased endothelial expression of ICAM-1 and consequently reduced monocyte adhesion to the vascular endothelium. In hypercholesterolemia, modified lipoproteins induce endothelium activation, thereby mediating the arrest and transmigration of circulating monocytes into the subendothelial space where they differentiate into macrophages (46).

In addition to monocyte adhesion, alirocumab reduced other markers of vascular inflammation, including T-cell abundance in the aortic root area, as well as macrophage and necrotic content and cholesterol clefts of the lesions. Cholesterol crystals have been shown to be particularly proinflammatory and to trigger local and systemic inflammatory responses (48, 49). Moreover, increased macrophage content and a large necrotic core, as well as a thin, collagen-poor fibrous cap and decreased SMCs, are important characteristics of a vulnerable lesion that is prone to rupture (45). Alirocumab alone and in combination with atorvastatin reduced vascular inflammation and strongly improved the plaque morphology.

The present study is a progression/prevention study that may pose a potential limitation with respect to translation to the clinic where patients with existing lesions are often treated. Nonetheless, data from this study may suggest beneficial effects on markers of atherosclerosis by reducing TC with alirocumab also in the human situation where new lesions are formed alongside existing plaques.

PCSK9 has received a considerable amount of attention in the past decade as a possible target for treatment of CVD, and several approaches to inhibit the protein are currently being investigated (4). Efficacy and safety of alirocumab will be further investigated in large phase 3 clinical outcome trials, in patients with FH and in high cardiovascular risk patients with hypercholesterolemia on lipid-modifying therapy within the ODYSSEY program (42). These trials will reveal whether PCSK9 inhibition with alirocumab translates into clinical benefit.

## Supplementary Material

Supplemental Data
