# Supplementary material for: Alirocumab inhibits atherosclerosis, improves the plaque morphology, and enhances the effects of a statin
Source: J Lipid Res. 2014 Oct;55(10):2103–12. doi: 10.1194/jlr.M051326 (PMC4174003; doi:10.1194/jlr.M051326)
Supplement: Supplemental Data [file supp_55_10_2103__index.html]

Alirocumab inhibits atherosclerosis, improves the plaque morphology, and enhances the effects of a statin — Alirocumab inhibits atherosclerosis, improves the plaque morphology, and enhances the effects of a statin — Alirocumab inhibits atherosclerosis, improves the plaque morphology, and enhances the effects of a statin — Supplemental Data 

# Alirocumab inhibits atherosclerosis, improves the plaque morphology, and enhances the effects of a statin

## Supplemental Data

**Files in this Data Supplement:**

- supplemental online data - Supplemental file with methods and results
