# Supplementary material for: Alirocumab inhibits atherosclerosis, improves the plaque morphology, and enhances the effects of a statin
Source: J Lipid Res. 2014 Oct;55(10):2103–12. doi: 10.1194/jlr.M051326 (PMC4174003; doi:10.1194/jlr.M051326)
Supplement: Supplemental Data [file supp_M051326_jlr.M051326-1.pdf]

Supplemental data

Methods

Animals

Ninety female APOE*3Leiden.CETP transgenic mice (9 to 13 weeks of age) (1), expressing human cholesteryl ester transfer protein under control of its natural flanking regions, were used. During the study, mice were housed under standard conditions with a 12-h light-dark cycle and had free access to food and water. Animal experiments were approved by the Institutional Animal Care and Use Committee of The Netherlands Organization for Applied Research.

Experimental design

Mice received a semi-synthetic cholesterol-rich diet, containing 15% (w/w) cacao butter and 0.15% cholesterol (Western-type diet [WTD]; Hope Farms, Woerden, The Netherlands) for a run-in period of 3 weeks to increase plasma total cholesterol (TC) levels up to ~15 mmol/l. Body weight (BW) and food intake were monitored regularly during the study. After matching based on BW, TC, plasma TG and age, mice (n = 15 per group) received a WTD alone or were treated with two dosages of alirocumab (3 or 10 mg/kg) alone or in combination with atorvastatin (3.6 mg/kg/d) for 18 weeks, and an arm with atorvastatin alone was added. Alirocumab (provided by Regeneron) was administered via weekly subcutaneous injections and atorvastatin was added to the diet. We aimed for a reduction in TC of about 20% to 30% with the dose of atorvastatin. At the end of the treatment period all animals were sacrificed by CO_2

inhalation. Livers and hearts were isolated to assess hepatic LDL receptor (LDLR) protein levels, lipid content, atherosclerosis development, and plaque composition.

Plasma lipids, lipoprotein analysis and measurement of alirocumab levels

Plasma was isolated from blood collected in EDTA-coated cups via tail vein bleeding after a 4-h fast every 2 to 4 weeks. Plasma TC and TG were determined using enzymatic kits according to the manufacturer’s protocols (cat. no. 1458216 and cat. no. 1488872, respectively; Roche/Hitachi) and average plasma TC and TG levels were calculated by total exposure over number of weeks. Lipoprotein profiles for TC were measured after lipoprotein separation by fast protein liquid chromatography after 4, 12, and 18 weeks of treatment (1). Alirocumab levels were measured by a human Fc enzyme-linked immunosorbent assay.

Hepatic LDLR protein levels

Liver tissues were homogenized in lysis buffer (50 mM Tris-HCL [pH=7.4], 150 mM NaCl, 0.25% deoxycholic acid, 1% NP-40 [Igepal], 1mM EDTA, protease inhibitor cocktail [complete, Roche], 1 mM PMSF, 1 mM Na3VO4) and then centrifuged at 6500 rpm at 4˚C for 30 min. Protein concentration in cell lysates was determined by bicinchonic acid protein assay (Thermo Scientific) according to manufacturer's instructions. 50 µg of protein lysates were separated by SDS-PAGE and then transferred to polyvinylidene fluoride membranes (Millipore). Blots were subjected to goat anti-mouse LDLR from R&D Systems and rabbit anti-goat horseradish peroxidase (HRP) from AbD Serotec or mouse anti-α-Tubulin from Sigma and horse anti-mouse HRP from Cell Signaling Technologies (according to the manufacturer's instructions); blots were developed with West Femto Super

Signal ECL (Thermo Scientific) and subjected to the Chemi-Doc-it imaging system. Intensities of protein bands were quantified using Image J software.

Hepatic lipid analysis and fecal excretion of bile acids and neutral sterols

Liver tissue samples were homogenized in phosphate-buffered saline, and the protein content was measured. Lipids were extracted, separated by high-performance thin-layer chromatography on silica gel plates, and analyzed with TINA2.09 software (Raytest Isotopen Messgeräte Straubenhardt, Germany), as previously described (2).

Mice were housed at five mice per cage, and feces were collected during two consecutive periods of 72 h and 48 h, respectively. Aliquots of lyophilized feces were used for determination of neutral and acidic sterol content by gas-liquid-chromatography, as previously described (3).

Histological assessment of atherosclerosis

To determine the total plaque load in the thoracic aorta, perfusion-fixed aortas (from the aortic origin to the diaphragm) were cleaned of extravascular fat, opened longitudinally, pinned en face, and stained for lipids with oil-red O as described previously (7). Data were normalized for analyzed surface area and expressed as percentage of the stained area. Photos/images were taken with the Olympus BX51 microscope and lesion areas were measured using Cell D imaging software (Olympus Soft Imaging Solutions).

In the aortic root, lesion composition was determined for the severe lesions (type IV-V) as a percentage of lesion area after immunostaining with mouse anti-human

alpha actin (1:800; Monosan, Uden, The Netherlands) for smooth muscle cells (SMC), and rat anti-mouse Mac-3 (1:25; BD Pharmingen, the Netherlands) for macrophages followed by sirius red staining for collagen (5, 6, 8). Rat anti-mouse CD54 antibody, GTX76543 (GeneTex, Inc., San Antonio, TX, USA) was used for immunostaining of intercellular adhesion molecule 1 (9). Photos/images of the lesions were taken with the Olympus BX40 microscope with Nuance 2 multispectral imaging system, and stained areas were quantified using Image J software.

Flow cytometric analysis

After 8 weeks of treatment, peripheral blood mononuclear cells (PBMCs) were isolated from fresh blood samples and were sorted into GR-1+ (neutrophils/granulocytes), GR-1- (lymphocytes/monocytes), CD3+ (T-cells), CD19+ (B-cells) and CD11b+/Ly6C^{low} and CD11b+/Ly6C^{hi} (monocytes) cells using flow cytometric analysis. The following conjugated monoclonal antibodies were used from Becton Dickinson: GR-1 FITC, CD3 PerCpCy5-5, CD19 V450, CD11b APC and Ly6C PE-Cy7.

Statistical analysis

Significance of differences between the groups was calculated non-parametrically using a Kruskal-Wallis test for independent samples, followed by a Mann-Whitney U-test for independent samples. Linear regression analyses were used to assess correlations between variables. Since the atherosclerotic lesion area showed a quadratic dependence on plasma cholesterol exposure, it was transformed using square root transformation.

IBM SPSS Statistics 20 for Windows (SPSS, Chicago, USA) was used for statistical analyses. All groups were compared to the control group and to the atorvastatin group, and 3 mg/kg alirocumab was compared to 10 mg/kg alirocumab either with or without atorvastatin. Values are presented as means ± SD. Bonferroni-Holm's method was used to determine the level of significance in the case of multiple comparisons. *P*-values <0.05 were considered statistically significant. In figures, significant effects after correction for multiple comparisons are indicated by * to compare to the control group, † to compare to the atorvastatin group and ‡ to compare 3 mg/kg alirocumab to 10 mg/kg alirocumab.

Results

Cross reactivity of alirocumab with mouse PCSK9 and immune response

Alirocumab binds both human and mouse PCSK9 with high affinity (Kd=0.58nM and 2.6nM, respectively, at pH 7.4 and 25°C) as determined by a surface plasmon resonance experiment performed on a BiaCore T100 instrument where the PCSK9 proteins were injected over alirocumab that was immobilized on the sensor surface through a covalently-coupled anti-human Fc-gamma-specific polyclonal antibody.

Since the mice were injected for a long period of time with a human monoclonal antibody, we were aware of potential immune responses and, therefore, tested this response for alirocumab in a pilot study in advance of the atherosclerosis study. In this pilot study no mouse –anti human antibody was detected for any of the doses tested. During the atherosclerosis study we closely monitored the animals. No immune response was observed as evidenced by stable efficacy throughout the study as demonstrated in figure F1.

Alirocumab does not affect hepatic lipids and fecal bile acid and neutral sterol excretion

To evaluate the consequences of alirocumab-induced alterations in lipoprotein metabolism on hepatic lipid metabolism and excretion into feces, we determined liver lipids and excretion of bile acids and neutral sterols in stool. Alirocumab did not affect liver weight nor the hepatic content of cholesterol and TG, whereas the combination of 10 mg/kg alirocumab and atorvastatin led to a significant reduction in liver weight (-20%, $P < 0.05$) and atorvastatin and the combination treatments led to significant reductions in hepatic cholesteryl esters (-48%, $P < 0.05$; -41%, $P < 0.05$

and -44%, p=0.28, respectively) as compared to the control group, without a change in hepatic TG (Table S1 and Table S2). Fecal output of bile acids and neutral sterols was not changed by the treatments (Table S3). These data indicate that despite the greater influx of cholesterol from the plasma compartment hepatic cholesterol homeostasis is maintained during alirocumab and statin treatment in mice.

Alirocumab reduces circulating monocytes

The effects of alirocumab alone and in combination with atorvastatin on white blood cell count was assessed by flow cytometry (Table S4). Interestingly, alirocumab alone and together with atorvastatin reduced granulocytes/neutrophils (-20%, $P < 0.05$; -34%, $P < 0.01$) and monocytes (-28%, $P < 0.05$; -39%, $P < 0.01$) when expressed as a percentage of the PBMC population. More specifically, alirocumab alone and in combination with atorvastatin tended to decrease pro-inflammatory Ly6C^{hi} (-8%, N.S.; -19%, $P < 0.001$) and increase anti-inflammatory Ly6C^{low} (+12%, N.S.; +35%, $P < 0.001$) monocytes. Therefore, the effect of alirocumab on vascular recruitment and adhesion of monocytes may be augmented by a reduction in circulating monocytes.

References

1. Westerterp, M., C. C. van der Hoogt, W. de Haan, E. H. Offerman, G. M. Dallinga-Thie, J. W. Jukema, L. M. Havekes, and P. C. Rensen. 2006. Cholesteryl ester transfer protein decreases high-density lipoprotein and severely aggravates atherosclerosis in APOE*3-Leiden mice. *Arterioscler. Thromb. Vasc. Biol.* **26:** 2552-2559.

2. Post, S. M., J. P. Zoeteweij, M. H. Bos, E. C. de Wit, R. Havinga, F. Kuipers, and H. M. Princen. 1999. Acyl-coenzyme A:cholesterol acyltransferase inhibitor, avasimibe, stimulates bile acid synthesis and cholesterol 7alpha-hydroxylase in cultured rat hepatocytes and in vivo in the rat. *Hepatology.* **30:** 491-500.

3. Post, S. M., R. de Crom, R. van Haperen, A. van Tol, and H. M. Princen. 2003. Increased fecal bile acid excretion in transgenic mice with elevated expression of human phospholipid transfer protein. *Arterioscler. Thromb. Vasc. Biol.* **23:** 892-897.

4. Delsing, D. J., E. H. Offerman, W. van Duyvenvoorde, H. van der Boom, E. C. de Wit, M. J. Gijbels, A. van der Laarse, J. W. Jukema, L. M. Havekes, and H. M. Princen. 2001. Acyl-CoA:cholesterol acyltransferase inhibitor avasimibe reduces atherosclerosis in addition to its cholesterol-lowering effect in ApoE*3-Leiden mice. *Circulation* **103:** 1778-1786.

5. Kuhnast, S., J. W. van der Hoorn, A. M. van den Hoek, L. M. Havekes, G. Liau, J. W. Jukema, and H. M. Princen. 2012. Aliskiren inhibits atherosclerosis development and improves plaque stability in APOE*3Leiden.CETP transgenic mice with or without treatment with atorvastatin. *J Hypertens.* **30:** 107-116.

6. Stary, H. C., A. B. Chandler, R. E. Dinsmore, V. Fuster, S. Glagov, W. Insull, Jr., M. E. Rosenfeld, C. J. Schwartz, W. D. Wagner, and R. W. Wissler. 1995. A definition of advanced types of atherosclerotic lesions and a histological classification of atherosclerosis. A report from the Committee on Vascular Lesions of the Council on Arteriosclerosis, American Heart Association. *Arterioscler. Thromb. Vasc. Biol.* **15:** 1512-1531.

7. Verschuren, L., R. Kleemann, E. H. Offerman, A. J. Szalai, S. J. Emeis, H. M. Princen, and T. Kooistra. 2005. Effect of low dose atorvastatin versus diet-induced cholesterol lowering on atherosclerotic lesion progression and inflammation in apolipoprotein E*3-Leiden transgenic mice. *Arterioscler. Thromb. Vasc. Biol.* **25:** 161-167.

8. Kuhnast, S., M. C. Louwe, M. M. Heemskerk, E. J. Pieterman, J. B. van Klinken, S. A. van den Berg, J. W. Smit, L. M. Havekes, P. C. Rensen, J. W. van der Hoorn, H. M. Princen, and J. W. Jukema. 2013. Niacin Reduces Atherosclerosis Development in APOE*3Leiden.CETP Mice Mainly by Reducing NonHDL-Cholesterol. *PLoS. One.* **8:** e66467.

9. Verschuren, L., J. de Vries-van der Weij, S. Zadelaar, R. Kleemann, and T. Kooistra. 2009. LXR agonist suppresses atherosclerotic lesion growth and promotes lesion regression in apoE*3Leiden mice: time course and mechanisms. *J Lipid Res* **50:** 301-311.

Tables

Table S1 Safety aspects of alirocumab**.**

	Safety aspects		
	**Plasma ALT (U/L)**	**Plasma AST (U/L)**	**Liver weight (g)**
Control	91.6 ± 75.6	223.0 ± 189.6	1.23 ± 0.25
3 mg alirocumab	67.1 ± 42.7	189.4 ± 108.7	1.18 ± 0. 25
10 mg alirocumab	153.0 ± 122.6	426.3 ± 309.2 *	1.38 ± 0.43
Atorvastatin	59.8 ± 24.3	217.0 ± 76.5	1.05 ± 0.12
3 mg alirocumab + atorvastatin	54.5 ± 25.6	171.7 ± 66.1	1.02 ± 0.17
10 mg alirocumab + atorvastatin	51.0 ± 23.7	157.6 ± 29.2 †	0.99 ± 0.10 *

ALT = alanine transaminase; AST = aspartate transaminase.

*$P < 0.05$, as compared to control, †$P < 0.05$ as compared to atorvastatin

Table S2 Effect of alirocumab, atorvastatin and their combination on liver lipids.

	Liver lipids (µg/mg protein)		
	**FC**	**CE**	**TG**
Control	11.6 ± 1.6	50.6 ± 14.0	119.2 ± 33.3
3 mg alirocumab	11.2 ± 1.4	48.2 ± 8.2 †	117.7 ± 21.6
10 mg alirocumab	11.4 ± 2.0	53.9 ± 10.4 †††	142.1 ± 43.0
Atorvastatin	9.5 ± 0.9	26.2 ± 4.8 *	90.6 ± 28.5
3 mg alirocumab + atorvastatin	10.4 ± 1.8	29.6 ± 5.8 *	103.5 ± 36.8
10 mg alirocumab + atorvastatin	10.7 ± 1.2	28.3 ± 9.0	109.8 ± 28.8

FC = free cholesterol; CE = cholesterol esters

*$P < 0.05$ as compared to control; †$P < 0.05$, †††$P < 0.001$ as compared to atorvastatin

Table S3 Effect of alirocumab, atorvastatin and their combination on neutral sterol and bile acid excretion.

	**Neutral sterol excretion (μmol/100 g mouse/day)**	**Bile acid excretion (μmol/100 g mouse/day)**
Control	25.8 ± 5.5	13.5 ± 3.3
3 mg alirocumab	20.4 ± 6.2	14.3 ± 2.7
10 mg alirocumab	21.6 ± 5.6	12.4 ± 3.2
Atorvastatin	30.3 ± 6.5	10.7 ± 2.4
3 mg alirocumab + atorvastatin	28.6 ± 6.0	11.4 ± 2.2
10 mg alirocumab + atorvastatin	27.5 ± 4.4	12.7 ± 1.6

Table S4 Effect of alirocumab, atorvastatin, and their combination on white blood cell count as assessed by flow cytometric analysis after 8 weeks of treatment.

	Control	10 mg/kg Alirocumab	Atorvastatin	10 mg/kg Alirocumab + Atorvastatin
**Neutrophils/granulocytes** (% of PBMC population)	8.9 ± 2.4	7.1 ± 2.0 * †	5.1 ± 1.6 ***	5.9 ± 2.0 **
**Lymphocytes/monocytes** (% of PBMC population)	91.1 ± 2.4	92.9 ± 2.0 * †	94.9 ± 1.6 ***	94.1 ± 2.0 **
▪ **T-cells** (% of PBMC population)	22.9 ± 4.6	21.4 ± 5.0 †	17.0 ± 4.8 **	18.5 ± 4.5 *P*=0.054
▪ **B-cells** (% of PBMC population)	63.9 ± 8.5	66.3 ± 15.0	69.5 ± 17.4 **	66.9 ± 2.0 **
▪ **Monocytes** (% of PBMC population)	12.3 ± 5.0	8.9 ± 2.5 * †††	5.3 ± 2.4 ***	7.5 ± 2.8 ** †
▪ ***CD11b+ Ly6C**^hi^* (% of monocytes)	62.2 ± 8.5	57.5 ± 8.4	51.2 ± 6.4 **	50.2 ± 4.1 ***
▪ ***CD11b+ Ly6C**^low^* (% of monocytes)	35.6 ± 7.7	40.0 ± 8.0 †	47.8 ± 6.3 ***	48.0 ± 3.5 ***

PBMC = peripheral blood mononuclear cells

*P < 0.05, **P < 0.01, ***P < 0.001 as compared to control; †P < 0.05, †††P < 0.001 as compared to atorvastatin (n = 15 per group)

Figures

Figure F1 Effect of alirocumab, atorvastatin and their combination on plasma cholesterol levels in time

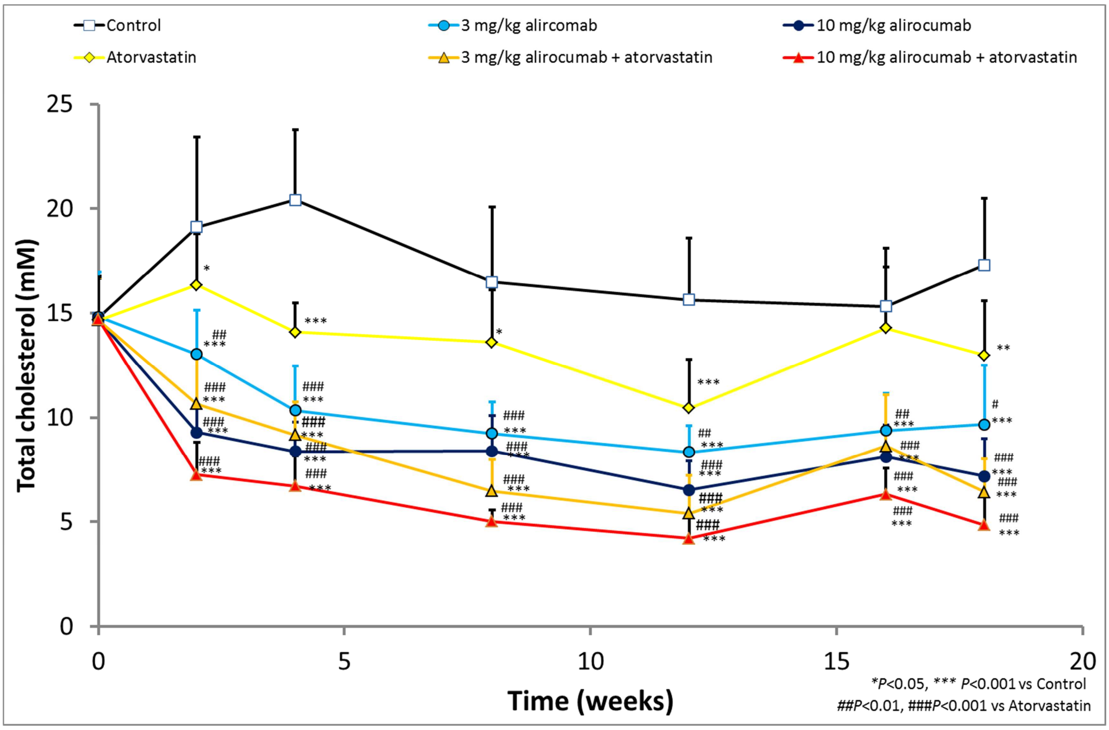

Values are mean +/- SD for 15 mice per group.
